# Estimation of care costs for individuals with cystic fibrosis at a referral center: cohort study, Salvador, 2005–2022

**DOI:** 10.1590/S2237-96222026v35e20250072.en

**Published:** 2026-02-23

**Authors:** Juliana Cana Brazil Costa, Ney Cristian Amaral Boa Sorte, Edna Lúcia D’Souza

**Affiliations:** 1 Universidade Federal da Bahia, Programa de Pós-graduação em Processos Integrativos dos Órgãos e Sistemas, Salvador, BA, Brazil; 2 Hospital Universitário Professor Edgard Santos, Núcleo de Avaliação de Tecnologias em Saúde, Salvador, BA, Brazil; 3 Universidade Federal da Bahia, Programa de Pós-graduação em Processos Integrativos dos Órgãos e Sistemas, Salvador, BA, Brazil

**Keywords:** Cystic fibrosis, Rare Diseases, Health Care Costs, Costs and Cost Analysis, Cohort Studies, Fibrosis Quística, Enfermedades Raras, Costos de la Atención en Salud, Costos y Análisis de Costo, Estudios de Cohortes

## Abstract

**Objectives:**

To estimate the direct costs of diagnosing and treating cystic fibrosis in individuals followed at a referral center in Salvador, and to compare treatment costs between individuals with early diagnosis and those with delayed diagnosis.

**Methods:**

Cohort study that included individuals followed from January 2005 to April 2022 and that used data obtained from the institution’s information system for outpatient data, and from the Hospital Unit Activity Generation System (SISAIH) for hospitalization data. A data collection approach using the micro-costing method was employed. Participants were classified into two groups: early diagnosis and delayed diagnosis. Care costs between groups were compared. The mean and standard deviation were reported for continuous variables with normal distributions, and the median and interquartile range for non-normal variables. The nonparametric Mann–Whitney U test was used to compare costs between the two groups. The correlation between costs was assessed by follow-up time in each group.

**Results:**

Data from 66 individuals were evaluated. Medication costs accounted for the majority (BRL 6,497,011.60). Patients with delayed diagnosis had a 1.5-fold higher cost ratio (BRL 11,167.45) than those with early diagnosis (BRL 7,294.77), with a statistically significant difference (p-value<0.001). There was a weak correlation between care costs and follow-up time for both early- and delayed-diagnosis groups.

**Conclusion:**

The costs of cystic fibrosis care were high, mainly those related to medication consumption for individuals with delayed diagnosis.

## Resumen

**Objetivos:**

Estimar los costos directos del diagnóstico y tratamiento de la fibrosis quística en individuos atendidos en un centro de referencia en Salvador y comparar los costos del tratamiento entre individuos con diagnóstico precoz y aquellos con diagnóstico tardío.

**Métodos:**

Estudio de cohorte que incluyó individuos atendidos entre enero de 2005 y abril de 2022, utilizando datos obtenidos del sistema de información de la institución para los datos ambulatorios y del Sistema Generador del Movimiento de las Unidades Hospitalarias (SISAIH) para los datos de hospitalización. Se utilizó un enfoque de recopilación mediante microcosteo. Los participantes se clasificaron en dos grupos: diagnóstico temprano y diagnóstico tardío. Se compararon los costos de atención entre los grupos. Se describieron la media y la desviación estándar para las variables continuas con distribución normal, y la mediana y el rango intercuartílico para las variables no normales. Se utilizó la prueba no paramétrica U de Mann-Whitney para comparar los costos entre los dos grupos. Se evaluó la correlación entre los costos y el tiempo de seguimiento en cada grupo.

**Resultados:**

Se evaluaron datos de 66 individuos. Los costos se concentraron principalmente en medicamentos (BRL 6.497.011,60). Los pacientes con diagnóstico tardío presentaron una razón de costos 1,5 veces mayor (BRL 11.167,45) en comparación con los de diagnóstico temprano (BRL 7.294,77), con diferencia estadísticamente significativa (valor p<0,001). Se observó una correlación débil entre los costos de atención y el tiempo de seguimiento tanto para los individuos con diagnóstico temprano como para aquellos con diagnóstico tardío.

**Conclusión:**

Los costos de la atención a la fibrosis quística fueron elevados, especialmente aquellos relacionados con el consumo de medicamentos en individuos con diagnóstico tardío.

**Ethical aspects**

This research used public domain anonymized databases.

## Introduction 

Cystic fibrosis is a rare genetic disease caused by pathogenic variants in the cystic fibrosis transmembrane conductance regulator (CFTR) gene (1,2), with substantial clinical heterogeneity due to wide variability in manifestations across affected organs and systems and in symptom severity (3). In Brazil, until 2021, 6,427 individuals with the disease had been diagnosed and were receiving care at 53 centers across the country, of whom 490 (8%) were in the state of Bahia (4). 

Worldwide, due to early diagnosis through newborn screening, improved access to multidisciplinary health services, and more effective treatments, reductions in complications, morbidity and mortality have been observed, along with a consequent increase in life expectancy (5,6,7). However, follow-up of these individuals has been associated with high economic costs (8). In Germany, in 2019, the mean direct medical costs were estimated at EUR 17,551.00 per patient per year, with medications accounting for the largest share (9).

CFTR protein modulators, which are precision medicines, unlike symptomatic therapy, correct or potentiate protein function, promoting significant clinical improvement. The incorporation of these medications, including in Brazil (10), will increase expenditures for the Brazilian Unified Health System (*Sistema Único de Saúde*, SUS). However, there is the prospect of reduced costs associated with hospitalizations and other outcomes linked to greater disease severity (7). The incremental budget impact was estimated to range from R$354 million to R$431 million per year, totaling R$1.99 billion between 2024 and 2028, for incorporating Elexacaftor/Tezacaftor/Ivacaftor (Trikafta) into SUS (11). 

With increased life expectancy resulting from these new treatments—generally cumulative technologies—prospects include higher medication expenditures, as well as reductions in hospitalizations and in the treatment of complications (7). This balance tends to favor increased expenditures due to the high cost of new therapies, which poses a major challenge to universal access to health in Brazil amid budget constraints (12).

This study aimed to estimate the direct costs of diagnosing and treating cystic fibrosis in individuals followed at a referral center in the state of Bahia. Additionally, treatment costs were compared between individuals with early and delayed diagnoses.

## Methods 

### Study design

This was an open ambispective cohort study, with retrospective (January 2005 to January 2021) and prospective (February 2021 to April 2022) components. Cohort admission was defined as the first evaluation at the institution, whether through outpatient consultation after diagnosis or hospital admission. Cohort exit occurred due to death, transfer of the individual to another center, treatment abandonment—characterized by absence of consultations for more than 12 months—or the end of data collection, which occurred on April 30, 2022. Regardless of the reason for exit, the contribution time in the cohort was accounted for and expressed as person-years. 

### Setting 

The study was conducted in a public university hospital located in Salvador, Bahia, which provides medium- and high-complexity inpatient and outpatient care. The hospital is a referral center for rare diseases and is accredited as one of the two cystic fibrosis referral services in the state, with an emphasis on pediatric and adolescent care and an average of four diagnoses per year (13). 

At the service, individuals aged 0–25 years were treated. Children under one year of age are evaluated monthly, and those older than one year are evaluated every three months or at a shorter interval if necessary. A multidisciplinary team conducted consultations, and regular complementary tests were requested, as established in the Clinical Protocols and Therapeutic Guidelines (*Protocolo Clínico e Diretrizes Terapêuticas*, PCDT) (14).

### Participants 

A purposive non-probability sample was selected based on: (i) confirmed diagnosis of cystic fibrosis, defined by elevated sweat chloride on two occasions and identification of two pathogenic variants in the CFTR gene; (ii) regular follow-up (having had two consultations in the previous year) at the service; and (iii) signature of the informed consent form. There were no exclusion criteria. Those who did not complete follow-up (losses) had their data included up to the moment of cohort exit. 

Participants were classified as those diagnosed based on clinical findings (delayed diagnosis) and those who underwent newborn screening with a positive result (early diagnosis).

### Variables 

Sociodemographic and clinical variables included age, sex, race/skin color, type of diagnosis, diagnostic tests used (sweat test), number of hospitalizations, and length of hospitalizations (in days). 

Direct outpatient costs were recorded (quantity and costs of regular consultations with medical and non-medical professionals), quantity and costs of medications, distributed according to the Bahia pharmaceutical assistance program (pancreatin, dornase alfa, and tobramycin), quantity and costs of outpatient diagnostic support tests (chest, abdominal, and other CT scans; pulmonary function assessment; abdominal and other ultrasound exams; bacteriological culture of oropharynx or sputum; chest and abdominal radiological exams), hospitalization costs, medication costs dispensed in outpatient care and during hospitalizations, and costs of patient meals, and linen and laundry services. 

### Data sources and payer perspective 

Sociodemographic and clinical data of individuals were obtained from paper and electronic medical records. 

Costs were estimated from two perspectives: (i) SUS as provider, considering funding transfers made to the hospital for outpatient consultations and examinations, disease-specific medications, and hospitalizations; and (ii) the health institution as a health service provider, referring to expenditures not covered by the SUS (Table 1).

**Table 1:** Detailed description of the sources and valuation of the costs assessed, according to cost type (medications, tests, consultations, hospitalizations, meals, and linen and laundry services) and payer perspective (Brazilian Unified Health System – SUS and healthcare institution). Salvador, 2022

Type of costs assessed	Perspective			
	SUS		Healthcare Establishment	
	Source	Note	Source	Note
**Medication**				
Cystic fibrosis protocol (dornase alfa, tobramycin, pancreatin)	SmartHealth	The recorded values correspond to the funding transfers from the Sesab^a^ by the Ministry of Health.	SmartHealth	Not included, as the institution does not incur these costs.
Other medications	Not applicable.	Not applicable.	SmartHealth	Acquisition costs through public bidding, updated for April 2021.
Tests	SIGTAP table^b^	Only outpatient tests were included.	SIGTAP table	Only tests conducted during hospitalizations that are not covered by SUS (such as laboratory tests and X-rays), as well as sweat chloride tests (from the third outpatient test onward or any test performed in hospitalized patients), were included.
Consultations	SIGTAP table	Consultations with medical and other health professionals were included.	Not applicable.	Consultations performed during hospitalizations were not included.
Admissions	SIGTAP table and paid AIH^c^.	Computed total values of AIH paid (includes value of the procedure, inpatient follow-up consultation, specific tests with allowed transfer, physiotherapy, use of blood and derivatives, some nutritional therapies, companion daily fee, ICU^d^ daily fee, wound dressings, surgical procedures)	Not applicable.	No specific costs were assessed.
Meals	Not applicable.	Not applicable.	Electronic public bidding	Daily meal costs were calculated separately.
**Linen and laundry services**	Not applicable.	Not applicable.	Electronic public bidding	Value defined per hospital linen kit. Six kits were provided per patient, with 80% of the kit costs transferred to the service provider.

^a^Sesab: Departemt of Health of the State of Bahia (*Secretaria de Saúde da Bahia*); ^b^SIGTAP: SUS Table of Procedures, Medicines, Orthotics, Prosthetics and Special Materials (*Sistema de Gerenciamento da Tabela de Procedimentos, Medicamentos e OPM do SUS*) ; ^c^AIH: Hospital Admission Authorization (*Autorização de Internação Hospitalar*); ^d^ICU: Intensive Care Unit.

For estimates of care costs, a bottom-up (micro-costing) approach was used, with identification of all resources used for each participant. Outpatient resources were obtained from the SmartHealth hospital information system, and inpatient resources from the Hospital Unit Activity Generation System (*Sistema Gerador do Movimento das Unidades Hospitalares*, SISAIH). All costs were collected at updated rates, based on values in Brazilian reais as of July 2022. 

### Study size

Of the 68 patients registered at the referral center, 66 (97%) were included. Two cases were excluded because they had fewer than two follow-up consultations at the time the study ended. 

### Operationalization of variables and statistical methods

Data were collected using standardized forms developed in Google Forms, tabulated in Microsoft Excel, and analyzed using the statistical packages R for Windows 4.2.2 and Stata for Mac 18.0.

To calculate total costs, the unit cost of a treatment was multiplied by the quantity used, resulting in an estimate of the average treatment cost. The mean direct cost was estimated for medications used during hospitalization, meals, and linen and laundry services. The costs assessed were expressed in monetary units (Brazilian reais), in total values, mean, and standard deviation, and per person-year. 

Costs were described by type (medications, consultations, tests, meals, and linen and laundry services), by location (hospitalization, inpatient, and outpatient), and by period (2005–2022 and 2013–2022). This latter category was used because participants with early diagnosis only began follow-up after the implementation of newborn screening in Bahia in 2013 (5,13).

The mean and standard deviation were reported for continuous variables with normal distributions, and the median and interquartile range for non-normal variables. For comparison of proportions and medians between those with delayed and early diagnosis, the chi-square test or Fisher’s exact test, when indicated, and the nonparametric Mann–Whitney U test were used. Spearman’s correlation test was used to analyze the association between follow-up time and cost. Correlation strength was classified as previously described (16). Given differences in cohort participation time between those with early versus delayed diagnosis, hospitalization density was calculated and expressed per 100 people-years. 

In all analyses, two-tailed tests and p-values less than or equal to 0.05 were considered statistically significant.

## Results 

A total of 66 participants were evaluated, of whom 47 (71.2%) had delayed diagnosis, identified from 2005 onward, and 19 (28.8%) had early diagnosis, born between 2013 and 2021, with males predominating (56.1%) (Table 2). The mean (standard deviation) age at cohort admission was 52.7 (±44.2) months. Diagnosis through newborn screening occurred earlier compared with those with delayed diagnosis (3.4±0.3 versus 72.0±10.4 months; p-value<0.001).

**Table 2:** Number of participants with cystic fibrosis, according to sociodemographic and clinical characteristics and diagnosis status at a university hospital. Salvador, 2005–2022 (n=66)

Variable	Total (n=66)	Early diagnosis (n=19)	Delayed diagnosis (n=47)
	n (%)	n (%)	n (%)
**Age at the end of the cohort** (years)			
0–9	37 (56.9)	19 (100.0)	18 (39.1)
10–19	21 (32.3)	-	21 (45.7)
≥20	7 (10.8)	-	7 (15.2)
Sex			
Female	29 (43.9)	9 (47.4)	20 (42.6)
Male	37 (56.1)	10 (52.6)	27 (57.4)
**Race/skin color**			
White	11 (16.7)	3 (15.8)	8 (17.0)
Brown (Brazilian mixed race)	48 (72.7)	14 (73.7)	34 (72.3)
Black	3 (4.5)	1 (5.3)	2 (4.2)
Not reported	4 (6.1)	1 (5.3)	3 (6.4)
**Need for hospitalization**			
No	24	6 (31.6)	18 (38.3)
Yes	42	13 (68.4)	29 (61.7)
Deaths			
No	62 (94.0)	17 (69.7)	45 (24.2)
Yes	4 (6.0)	2 (3.0)	2 (3.0)

The median (p25; p75) follow-up time was 95.9 (53.8; 173.9) months, ranging from 7.4 to 279.0 months. Participants with early diagnosis had a significantly shorter accumulated total follow-up time, in years, than those with delayed diagnosis (69.9 months vs. 550.8 months; p-value<0.001).

A total of 121 hospitalization episodes were identified: 96 (79.3%) occurred among participants with delayed diagnosis; of these, 61.7% (29/47) were hospitalized. Among those with early diagnosis, 68.4% (13/19) were hospitalized at some point during follow-up. Those with early diagnosis were predominantly hospitalized during the first year of life (88.0%) and had a median length of stay similar to those with delayed diagnosis. Hospitalization density (per 100 person-years) was 18.0 for those with delayed diagnosis and 34.3 for those with early diagnosis (p-value 0.008). The median (p25; p75) number of hospitalizations did not differ between those with early and delayed diagnosis (1.0 [0.0; 2.0]; range: 0–6 vs. 2.0 [0.0; 4.0]; range: 0–11; p-value 0.347).

In the period 2005–2022, the total cost (diagnosis and treatment) for all 66 participants, from the SUS perspective, was BRL 6,702,599.30, yielding a mean expenditure per patient-year of BRL 10,731.82. Values of BRL 7,294.77 per patient-year were observed for those with early diagnosis and BRL 11,167.45 per patient-year for those with delayed diagnosis, with a 1.5-fold higher cost ratio for patients with delayed diagnosis (p-value<0.001).

Direct medication costs accounted for 96.9% of total expenditures, totaling BRL 6,497,624.07 (Table 3). Dornase alfa accounted for the highest expenditures and had a higher value among those with delayed diagnosis (BRL 8,594.85 per patient-year) than among those with early diagnosis (BRL 3,253.43; p-value<0.001) (Table 3). Expenditures per person-year for those with delayed diagnosis, computed between 2013 and 2022, did not differ from the overall period (2005–2022), except for higher values for tobramycin (BRL 1,195.80 vs. BRL 554.24; p-value<0.001), outpatient consultations (BRL 82.26 vs. BRL 57.58; p-value<0.001), outpatient exams (BRL 81.49 vs. BRL 54.83; p-value<0.001), medications during hospitalization (BRL 159.32 vs. BRL 69.97; p-value<0.001), and patient meals during hospitalization (BRL 431.08 vs. BRL 272.09; p-value<0.001) (Table 3). 

**Table 3:** Total costs (BRL), mean and standard deviation (BRL), and per patient–year (BRL) for outpatient and hospital costs, by type, and incidence density ratios (IDR) of costs per patient-year, between individuals with early and delayed diagnosis, stratified by analysis period (2005–2022 and 2013–2022), from the perspectives of the Brazilian Unified Health System (SUS) and the healthcare institution. Salvador, 2005–2022 (n=66)

Costs in BRL (Type)	All cases (n=66)	2005–2022				IDR	All cases (n=66)	2013a–2022				IDR
		n	Delayed diagnosis	n	Early diagnosis			n	Delayed diagnosis	n	Early diagnosis	
Outpatient												
Medications^c^		45		19				17		19		
Total cost	6,497.624.07		6,042,576.53		455,047.54		1,666,038.00		1,210,990.00		455,047.50	
Mean±SD^b^	96,976.19±108,553.22		128,560.79±113,883.41		23,949.87 ±36,149.47		45,028.05±60,939.39		71,234.72±74,171.83		23,949.87±36,149.47	
Person-year	10,403.28		10,902.01		6,471.91	0.59	9,197.49		10,926.63		6,471.91	0.59
**Dornase alfa** ^c^		37		6				9		6		
Total cost	4,992,384.37		4,763,630.92		228,753.45		1,114,468.75		885,715.30		228,753.45	
Mean±SD	74,513.20 ±89,986.39		101,353.85 ±93,799.57		12,039.66±29,949.90		30,120.78±51,375.82		52,100.90±63,019.42		12,039.66±29,949.90	
Person-year	7,993.52		8,594.85		3.253.43	0.38	6,152.51		7,991.71		3,253.43	0.41
Tobramycin^c^		34		14				14		14		
Total cost	535,176.20		472,787.70		62,388.50		194,917.60		132,529.10		62,388.50	
Mean±SD	7,987.70±12,480.27		10,059.31±14,207.56		3,283.60±4,141.33		5,268.04±7,370.45		7,795.83±9,472.79		3,283.60±4,141.33	
Person-year	624.55		554.24		887.31	1.04	1,076.05		1,195.80		887.31	0.74
Pancreatin^c^		41		18				17		18		
Total cost	970,063.50		806,157.91		163,905.59		356,651.30		192,745.80		163,905.59	
Mean±SD	14,475.29±14,876.52		17,147.63±15,663.63		86,262.61 ±10,866.23		9,639.23±10,217.38		11,337.99±9,589.75		8,6262.61±10,866.23	
Person-year	1,552.86		1,454.13		2,331.15	1.60	1,968.93		1,739.13		2,331.15	1.34
Consultations^c^		47		19				17		19		
Total cost	37,876.50		31,913.40		5,963.40		15,080.80		9,117.40		5,963.40	
Mean±SD	563.33±394.50		679.01±400.96		313.86±203.83		443.55±235.18		536.32 ±250.63		313.86 ±203.83	
Person-year	60.64		57.58		84.81	1.47	83.25		82.26		84.81	1.03
Examinations^c^		45		19				16		19		
Total cost	40,317.03		30,391.15		9,925.88		18,958.08		9,032.20		9,925.88	
Mean±SD	601.75±431.77		646.62 ±488.52		522.41 ±204.05		512.38±251.30		531.31±279.89		522.41±204.05	
Person-year	64.55		54.83		141.17	2.57	104.66		81.49		141.17	1.73
Hospital												
Admissions^c^		27		13				11		13		
Total cost	126,781.40		84,813.64		41,967.77		73,452.58		31,484.81		41,967.77	
Mean±SD	1,173.90±1,478.33		1,804.55±2,670.26		2,208.83±4,447.06		1,985.20±3,494.46		1,850.05±2,214.11		2,208.83±4,447.06	
Person-year	203.00		153.03		596.89	3.91	405.50		284.09		596.89	2.10
Medication^d^		26		12				11		12		
Total cost	58,473.07		38,782.71		19,690.36		37,347.57		17,657.21		19,690.36	
Mean±SD	872.73±2,215.72		825.16 ±2,113.35		1,036.33±2,551.68		1,009.39±2,358.46		1,038.66±2,263.81		1,036.33±2,551.68	
Person-year	93.62		69.97		280.04	4.00	206.18		159.32		280.04	1.76
Meals^d^		27		13				11		13		
Total cost	191,621.70		150,804.70		40,816.98		88,592.58		47,775.60		40,816.98	
Mean±SD	2,860.03±4,138.92		3,208.61±4,693.54		2,148.26±2,282.31		2,394.39±2,694.80		2,810.33±3,140.90		2,148.26±2,282.31	
Person-year	306.81		272.09		580.52	2.13	489.08		431.08		580.52	1.35
**Linen and laundry services** ^d^		27		13				11		13		
Total cost	161,924.60		141,925.70		19,998.91		62,811.08		42,812.17		19,998.91	
Mean±SD	2,416.78±4,121.07		3,019.69±4,728.59		1,052.57±1,375.72		1,697.60±2,471.87		1,052.57±1,375.72		1,052.57±1,375.72	
Person-year	259.27		256.07		284.43	1.11	346.75		386.29		284.43	0.74

^a^Year of implementation of newborn screening for cystic fibrosis in Bahia, with costs for screened individuals collected from this period onward; ^b^SD: standard deviation; ^c^Cost components from the perspective of the Brazilian Unified Health System (SUS); ^d^Cost components from the perspective of the healthcare institution.

Outpatient costs for consultations and exams totaled BRL 80,533.10 (1.2% of total) (Table 3). A total of 5,029 consultations were recorded at the referral center, with per-patient costs ranging from BRL 22.60 to BRL 1,391.20, with a mean of BRL 565.00±BRL 371.00. A total of 215 imaging exams were performed in the outpatient setting and 136 during hospitalizations. A total of 1,491 respiratory bacteriology tests were conducted, with a total cost of BRL 15,282.75 (Table 4). 

**Table 4:** Quantity of health resource cost components used in the care of individuals with cystic fibrosis and their unit and total costs from the perspective of the Brazilian Unified Health System (SUS) at the university hospital. Salvador, 2005–2022 (n=66)

Cost components	Unit cost (BRL)	n	Total costs (BRL)
Outpatient			
Medical consultations	10.00	1,674	16,740.00
Consultations with non-medical professionals	6.30	3,355	21,136.50
**Diagnostic support tests** (outpatient)			
Assessment of lung function	9.14	25	228.50
Sweat chloride test	150.00	61	9,150.00
Echocardiogram	67.86	1	67.86
Gastrointestinal endoscopy	63.31	6	379.86
Bacteriological examination	10.25	1,491	15,282.75
Other CT scans	86.75	12	1,041.00
Other ultrasounds	24.20	11	266.20
Abdominal X-ray	10.73	1	10.73
Chest X-ray	9.50	91	864.50
Abdominal ultrasound	37.95	61	2,314.95
Doppler ultrasounds	39.60	4	158.40
Chest CT scan	136.41	25	3,410.25
Abdominal CT scan	277.26	3	831.78
Hospitalization^a^			
Admissions		121	126,781.41

^a^In the hospitalization costs, tests with values covered by the Brazilian Unified Health System (SUS) in Brazilian reais were included.

The total amount reimbursed by the SUS for sweat chloride testing was BRL 9,150.00 (Table 4). Between the first and second sweat chloride measurements, 112 tests were performed at the institution. From 2013 onward, SUS funding transfers to the institution covered the cost of up to two sweat chloride tests per patient, accounting for 54.5% of all tests performed. Eight individuals underwent their first sweat chloride measurement at another institution, and these were not counted. 

From the health facility’s perspective, total inpatient costs for medications, patient meals, and linen and laundry services amounted to BRL 412,019.37. From the SUS perspective, funding transfers for hospitalizations totaled BRL 126,781.40, accounting for 30.8% of the overall amount (Table 4). There was no statistically significant difference in these costs between patients with early and delayed diagnoses (p-value 0.416). Regarding medications prescribed during hospitalizations, there was no difference in the mean (±standard deviation) hospitalization costs per individual for those with early and delayed diagnosis (BRL 825.16±BRL2,113.35 vs. BRL 1,036.33±BRL 2,551.68; p-value 0.681).

**Figure 1:**
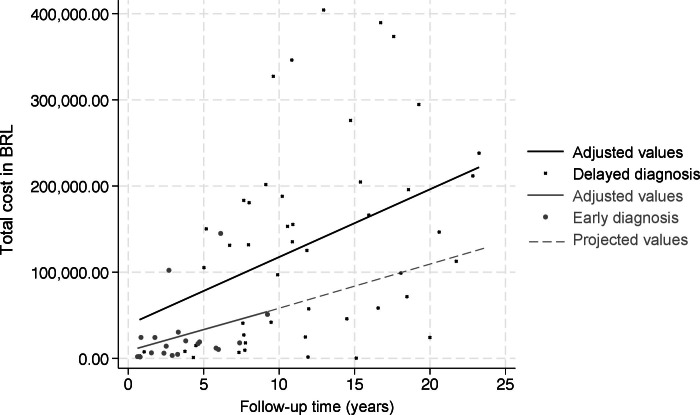
Correlation between total costs and follow-up time for participants with delayed diagnosis (n=47) and screened participants (n=18) at the university hospital. Salvador, 2005–2022

A weak correlation was observed between total costs and longer follow-up time, both for the group with delayed diagnosis (rho=0.324) and for those with early diagnosis (rho=0.313) (Figure 1).

There was a loss of 10 participants, with one transferred to another reference center, four deaths, and five due to loss to follow-up. The comparison between these 10 participants and the remaining participants did not reveal significant differences in follow-up duration (10.8±2.7 vs. 9.2±0.8; p-value 0.654) or length of hospitalization (18.1±9.4 vs. 29.4±5.5; p-value 0.125). Total costs (BRL 29,550.30±BRL 46,748.88 vs. BRL 113,741.10±BRL 114,722.50; p-value 0.003) and per person-year costs (BRL 3,143.96±BRL 2,602.54 vs. BRL 11,884.47±BRL 10,227.07; p-value 0.005) were significantly lower among those lost to follow-up.

The costs of 14 hospitalizations for 7 participants could not be measured due to insufficient detail in the SUS records. It was not possible to quantify the medications used by 6 participants with delayed diagnosis between 2005 and 2011, as the institution did not distribute these medications at that time. All other costs related to these individuals were included.

## Discussion 

The data demonstrated substantial investment by the SUS in the care of individuals with cystic fibrosis during 2005–2022. It was observed that medications accounted for 97% of the total expenditures, with lower spending per person-year among those with early diagnosis, at a ratio of 0.59 (medications), mainly due to the lower use of dornase alfa, but a higher ratio for expenditures on tests (1.73) and hospitalizations (2.10). 

The data showed the SUS commitment to comprehensive cystic fibrosis care but highlighted the need to review resource allocation, particularly given the high cost of medications. The greater use of tests and hospitalizations among early-diagnosis cases suggested the need for more intensive initial follow-up, which may prevent future complications. This reinforces the importance of early-detection policies and integrated care, aiming to sustain the system and improve individuals’ quality of life.

This study had some limitations. The single-center nature and the predominance of participants under 18 years of age limited the external validity of the results. However, in 2021, 75% of people with cystic fibrosis in Brazil were in this age group (4). Because it is a rare disease in the country, cost-analysis studies are scarce, as health institutions, especially public ones, do not track their costs and lack computerized systems to record expenditures for the health care provided (17). The institution lacked a cost center for disposable materials per individual, making it impossible to assess the supplies used and the associated fixed costs fully. 

Indirect costs and costs from other institutions were also not included, which limited the generalization of the findings and may have led to an underestimation of the average annual cost per patient. The values obtained were not validated with other hospitals or external sources. In fact, medications purchased through bidding processes may present large price variations depending on the amount purchased and the purchasing entity (state health department, health facility, federal government) (18). 

Another limitation was that the per–person–year costs were not adjusted for disease severity and age at diagnosis, potential confounders (19). Disease severity data were not collected in this study, and this is recommended for future research on this topic.

Previous studies showed that medications accounted for the largest share of costs associated with treating the clinical manifestations of the disease, reflecting dependence on continuous, high-cost therapies (20–23). Despite methodological differences across studies, there is consistency in the pattern of spending distribution, with medications and hospitalizations as the main components of total cost (6,23,24). This cost profile tends to intensify with disease progression, as expected in chronic conditions requiring highly complex therapeutic management (22). However, comparability of findings is limited by differences in the clinical protocols adopted, access to health technologies, and methodologies used to estimate costs across different national contexts (7,19,24).

In this study, dornase alfa was the medication with the greatest impact on per–patient–year cost, although it was significantly lower among screened individuals, as it is used only when there is potential benefit for lung function or risk of a lower respiratory tract infection (14,26). This finding reinforces what has been previously described: worse lung function is associated with higher cost (19). Considering only the period 2013–2022, the cost of tobramycin use was 35% higher among those with delayed diagnosis in this study.

Early and optimized treatment is expected to reduce costs by slowing disease progression. Lower demand for high-cost medications and avoidance of prolonged hospitalizations requiring antimicrobial therapy and oxygen supplementation support this hypothesis (27). This reinforces the role of newborn screening in reducing costs and maximizing outcomes. 

Previous studies may explain the lower per–patient–year cost observed among those with early diagnosis. In France, 779 children with cystic fibrosis born between 2006 and 2011 were followed for 10 years. It was observed that, in the first year of life, the average cost per patient was lower among early-diagnosis cases than among delayed-diagnosis cases based on symptoms (EUR 12,056.00±EUR 10,073.00; and EUR 3,861.00±EUR 17,493.00) (19). 

Three cost profiles were identified, with the majority of early-diagnosis individuals classified in the “low and stable” profile (19). This reinforces that confounding variables not assessed in this study may further strengthen this advantage. Possibly, if diagnostic confirmation and initiation of treatment for early-diagnosis cases occurred even earlier than the median described here, most hospitalizations of these children—which in this study were responsible for nearly nine out of ten hospitalizations in the first year of life—could have been avoided, reducing cost and, likely, future morbidity. Regarding observations in France, the “very high and increasing” cost profile was associated with worse pulmonary function, malnutrition, and increased infections with *Pseudomonas aeruginosa* (19).

Hospitalizations due to cystic fibrosis occur mainly because of respiratory infections, which are treated similarly between groups with early and delayed diagnosis, contributing to comparable costs. However, the shorter follow-up time in early-diagnosis patients and the concentration of hospitalizations in the first year suggest a possible cohort effect that may mask the economic benefits of newborn screening, which are expected to become more evident with longer follow-up, with fewer hospitalizations due to lower clinical severity. Studies have confirmed that hospital costs are associated with disease severity. In Canada in 2021, individuals with early diagnosis had better clinical outcomes and a lower need for hospitalization (28), a finding not reproduced in this analysis.

Although early diagnosis is essential to reduce morbidity and mortality, it does not ensure lower healthcare costs, especially in severe forms. Strengthening not only newborn screening but also the referral process and the earlier initiation of specialized treatment is important to expand clinical and economic benefits (13,27). This improvement is independent of the incorporation of new protein modulators, which have been approved for use in individuals aged six years or older (10). The efficiency of expenditures for the diagnosis and treatment of the disease depends on the incorporation of new medications and improvements in newborn screening (13).

No statistical difference in hospitalization costs was observed between individuals with early and delayed diagnosis. Hospitalization density was higher among those with early diagnosis, possibly due to the shorter follow-up time of these children in the cohort and the early identification of more severe cases, who might not survive without newborn screening. 

A hypothesis was raised that early diagnosis may have identified more severe cases that could have died before clinical diagnosis in the absence of newborn screening. In this study, 38.9% of the children diagnosed early had very prolonged hospitalizations at the time of diagnosis, which confirmed their clinical severity and a potential delay in diagnosis, even among screened children. In Bahia, delays in cystic fibrosis diagnosis through newborn screening were observed, with a median age at diagnosis of 3.5 months (13). This combination of factors likely maximized hospitalization density and prolonged hospital stays among those with early diagnosis, thereby introducing a cohort bias. Future analyses of this same cohort, considering the occurrence of clinical stability—achieved with adequate follow-up and reflected in the absence of hospitalizations—should reverse these findings.

The analysis from the perspective of the health institution showed an insufficient funding transfer percentage by SUS to cover hospitalization expenditures, including laboratory tests, medications, meals, and linen and laundry services, for which there is no direct funding transfer (29), regardless of the type and number of tests performed, thus failing to reflect the actual costs of hospitalizations. This reveals that the rational use of resources by care teams and greater efficiency in hospital care management for these individuals must be maximized (24,30). It also explains that cost analyses used in economic models from the SUS perspective underestimate costs, since all amounts not transferred to the hospital are covered with public resources. 

The study showed that micro-costing enabled accurate identification of the main cost components of cystic fibrosis, particularly medication costs. The detailed assessment identified the institution’s real costs, highlighting the insufficiency of public funding transfers and the need for greater efficiency in resource allocation (23;28-30)—newborn screening, when well performed, proved cost-effective by reducing complications and associated costs. 

The analyzed data indicated economic and social benefits of screening, such as reduced family suffering and a shorter diagnostic journey. Patients with delayed diagnosis presented higher direct costs per person-year, especially for medications required for more severe conditions. The findings of this study reinforced the need to review SUS funding transfers and to improve the efficiency of public program management.

## Data Availability

Data available upon request to the corresponding author.
