# Supplementary material for: Estimation of care costs for individuals with cystic fibrosis at a referral center: cohort study, Salvador, 2005–2022
Source: Epidemiol Serv Saude. 2026 Feb 23;35:e20250072. doi: 10.1590/S2237-96222026v35e20250072.en (PMC12928546; doi:10.1590/S2237-96222026v35e20250072.en)
Supplement: Translation

# Translation: Estimativa dos custos da assistência a indivíduos com fibrose cística em centro de referência: estudo de coorte, Salvador, 2005-2022

**DOI:** 10.1590/S2237-96222026v35e20250072.pt

**Authors:** Juliana Cana Brazil Costa, Ney Cristian Amaral Boa Sorte, Edna Lúcia D’Souza

**Affiliations:** 1 Universidade Federal da Bahia, Programa de Pós-graduação em Processos Integrativos dos Órgãos e Sistemas, Salvador, BA, Brasil; 2 Hospital Universitário Professor Edgard Santos, Núcleo de Avaliação de Tecnologias em Saúde, Salvador, BA, Brasil; 3 Universidade Federal da Bahia, Programa de Pós-graduação em Processos Integrativos dos Órgãos e Sistemas, Salvador, BA, Brasil

**Keywords:** Fibrose Cística, Doenças Raras, Custos de Cuidados de Saúde, Custos e Análise de Custo, Estudos de Coortes

## Abstract

**Objetivos:**

Estimar os custos diretos do diagnóstico e do tratamento da fibrose cística em indivíduos acompanhados em um centro de referência em Salvador e comparar os custos do tratamento entre os indivíduos com diagnóstico precoce e aqueles com diagnóstico tardio.

**Métodos:**

Estudo de coorte que incluiu indivíduos acompanhados entre janeiro de 2005 e abril de 2022 e que utilizou dados obtidos a partir do sistema de informação da instituição, para os dados ambulatoriais, e do Sistema Gerador do Movimento das Unidades Hospitalares, para os dados das internações. Utilizou-se abordagem de coleta pelo método de microcusteio. Os participantes foram classificados em dois grupos: diagnóstico precoce e diagnóstico tardio. Compararam-se os custos assistenciais entre os grupos. Foram descritas média e desvio-padrão, para as variáveis contínuas com distribuição normal, e mediana e intervalo interquartílico, para as não normais. Utilizou-se o teste não paramétrico U de Mann-Whitney para comparar os custos entre os dois grupos. Avaliou-se correlação entre os custos, conforme o tempo de seguimento em cada grupo.

**Resultados:**

Foram avaliados dados de 66 indivíduos. Predominaram custos com medicamentos (R$ 6.497.011,60). Pacientes com diagnóstico tardio apresentaram razão de custo 1,5 vez maior (R$ 11.167,45) em comparação aos com diagnóstico precoce (R$ 7.294,77), com diferença estatisticamente significativa (p-valor<0,001). Houve correlação fraca entre os custos assistenciais e o tempo de seguimento para os com diagnóstico precoce e para aqueles com diagnóstico tardio.

**Conclusão:**

Os custos da assistência à fibrose cística foram elevados, principalmente aqueles relacionados ao consumo de medicamentos para os indivíduos com diagnóstico tardio.

**Aspectos éticos**

Esta pesquisa utilizou bancos de dados de domínio público e anonimizados.

## Introdução 

A fibrose cística é uma doença genética rara, que decorre da presença de variantes patogênicas no gene *cystic fibrosis transmembrane conductance regulator* (CFTR) (1,2), com grande heterogeneidade clínica, tanto pela ampla variabilidade das manifestações nos órgãos e sistemas acometidos quanto pela gravidade dos sintomas (3). No Brasil, até 2021, foram diagnosticados 6.427 indivíduos com a doença, assistidos em 53 centros em todo o país, dos quais 490 (8%) foram na Bahia (4). 

Mundialmente, devido ao diagnóstico precoce pela triagem neonatal, ao melhor acesso a serviços de saúde multidisciplinares e aos tratamentos mais efetivos, observaram-se a redução das complicações, da morbimortalidade e, consequente, a elevação na expectativa de vida (5,6,7). Entretanto, o acompanhamento desses indivíduos associou-se a custos econômicos elevados (8). Na Alemanha, em 2019, foi estimada a média de custos médicos diretos de € 17.551,00 por paciente ao ano, com maior participação dos medicamentos (9).

Os moduladores da proteína CFTR, medicamentos de precisão, ao contrário da terapia sintomática, corrigem ou potencializam a função da proteína, o que promove importante melhora clínica. A incorporação desses medicamentos, inclusive no Brasil (10), ocasionará aumento dos gastos para o Sistema Único de Saúde (SUS), embora exista a perspectiva da redução de custos relativos aos internamentos e outros desfechos associados à maior gravidade da doença (7). Estimou-se impacto orçamentário incremental variando de R$ 354 a R$ 431 milhões, por ano, e R$ 1,99 bilhão, entre 2024 e 2028, para a incorporação do Elexacaftor/Tezacaftor/Ivacaftor (Trikafta) para o SUS (11). 

Com o aumento da expectativa de vida com esses novos tratamentos, por serem tecnologias, geralmente, cumulativas, as perspectivas são de aumento dos gastos com medicamentos, mas também de redução com internamentos e tratamento das complicações (7). Esse balanço tende a ser favorável ao aumento dos gastos, pelo elevado preço das novas terapias, o que representa grande desafio no acesso universal à saúde no Brasil diante das restrições orçamentárias (12).

Este estudo objetivou estimar os custos diretos do diagnóstico e do tratamento da fibrose cística em indivíduos acompanhados em um centro de referência no estado da Bahia. Adicionalmente, compararam-se os custos do tratamento entre os indivíduos com diagnóstico precoce e aqueles com diagnóstico tardio.

## Métodos 

### Delineamento do estudo

Trata-se de coorte ambispectiva aberta, com vertentes retrospectiva (janeiro de 2005 a janeiro de 2021) e prospectiva (fevereiro de 2021 a abril de 2022). A admissão na coorte foi o momento da primeira avaliação na instituição por consulta ambulatorial, após o diagnóstico, ou internamento hospitalar. A saída da coorte ocorreu por óbito, transferência do indivíduo para outro centro, abandono do tratamento, caracterizado por ausência de consultas por mais de 12 meses, ou término da coleta de dados, ocorrido em 30 de abril de 2022. Independentemente do motivo da saída, o tempo de contribuição na coorte foi considerado e expresso em pessoa-ano. 

### Contexto 

O estudo foi realizado em hospital público universitário, localizado em Salvador, Bahia, que presta assistência hospitalar e ambulatorial, de média e alta complexidades. O hospital é referência em doenças raras e habilitado como um dos dois serviços de referência em fibrose cística no estado, com ênfase no atendimento do público infantojuvenil, com média de quatro diagnósticos anuais (13). 

No serviço, foram atendidos indivíduos de 0-25 anos. Os menores de 1 ano de idade são avaliados mensalmente, e os maiores de 1 ano, a cada três meses ou em intervalo menor, se necessário. Realizaram-se consultas por equipe multiprofissional, sendo solicitados exames complementares regulares, conforme estabelecido no Protocolo Clínico e Diretrizes Terapêuticas (14).

### Participantes 

Amostra não probabilística intencional foi selecionada por: (i) diagnóstico confirmado de fibrose cística, definido pela elevação de cloreto no suor em duas ocasiões e identificação de duas variantes patogênicas no gene CFTR; (ii) acompanhamento regular (ter feito duas consultas no último ano) no serviço; e (iii) assinatura do registro de consentimento livre e esclarecido. Não houve critérios de exclusão. Aqueles que não completaram o seguimento (perdas) tiveram os dados incluídos até o momento de saída da coorte. 

Os participantes foram classificados entre aqueles com diagnóstico da doença com base em achados clínicos (diagnóstico tardio) e aqueles submetidos à triagem neonatal com resultado positivo (diagnóstico precoce).

### Variáveis 

As variáveis sociodemográficas e clínicas foram: idade, sexo, raça/cor da pele, tipo de diagnóstico, exames utilizados para o diagnóstico (teste do suor), número de hospitalizações e duração das hospitalizações (em dias). 

Registraram-se custos diretos ambulatoriais (quantidade e custos das consultas regulares dos profissionais médicos e não médicos), quantidade e custos de medicamentos, distribuídos conforme o programa de assistência farmacêutica da Bahia (pancreatina, alfadornase e tobramicina), quantidade e custos dos exames de apoio diagnóstico ambulatoriais (exames de tomografias de tórax, abdome e outras, avaliação da função pulmonar, ultrassonografia de abdome e outras, cultura bacteriológica de orofaringe ou escarro, exames radiológicos de tórax e abdome), custos dos internamentos, custos de medicamentos dispensados ambulatorialmente e durante os internamentos, custos com dieta do indivíduo e com rouparia. 

### Fontes dos dados e perspectiva do pagador 

Obtiveram-se os dados sociodemográficos e clínicos dos indivíduos, a partir dos prontuários físicos e eletrônicos. 

Estimaram-se os custos sob duas perspectivas: (i) do SUS como provedor, considerando-se os repasses realizados ao hospital pelas consultas e exames ambulatoriais, medicamentos específicos para o tratamento da doença e dos internamentos; e (ii) da instituição de saúde, como órgão prestador de serviços de saúde, referentes aos gastos não repassados pelo SUS (Tabela 1).

**Tabela 1:** Detalhamento das fontes e da valoração dos custos apurados, segundo tipo de custo (medicamentos, exames, consultas, internamentos, dieta, lavanderia) e perspectiva do pagador (Sistema Único de Saúde – SUS e estabelecimento de saúde). Salvador, 2022

Tipo dos custos aferidos	Perspectiva			
	SUS		Estabelecimento de Saúde	
	Fonte	Observação	Fonte	Observação
**Medicamentos**				
Protocolo de fibrose cística (alfadornase, tobramicina, pancreatina)	SmartHealth	Os valores cadastrados correspondem ao valor de repasse da Sesab^a^ pelo Ministério da Saúde	SmartHealth	Não foram computados, pois os valores não são gastos pela instituição
Outros medicamentos	Não se aplica	Não se aplica	SmartHealth	Valores de aquisição por meio dos pregões atualizados para abril de 2021
Exames	Tabela SIGTAP^b^	Computados somente exames ambulatoriais	Tabela SIGTAP	Computados somente os exames realizados durante os internamentos que não são repassados pelo SUS (análises clínicas, raio X) e teste do suor (a partir do terceiro teste ambulatorial ou qualquer teste em paciente internado)
Consultas	Tabela SIGTAP	Computadas consultas médicas e de outros profissionais de saúde	Não se aplica	Não foram computadas as consultas realizadas durante os internamentos
Internamentos	Tabela SIGTAP e espelhos das AIH^c^ pagas	Computados valores totais de AIH pagas (inclui valor do procedimento, consulta de acompanhamento de paciente internado, exames específicos com repasse permitido, fisioterapia, uso de sangue e derivados, algumas terapias nutricionais, diárias de acompanhante, diárias de UTI^d^, curativos, procedimentos cirúrgicos)	Não se aplica	Não foram apurados custos específicos
Dieta	Não se aplica	Não se aplica	Pregões eletrônicos	Valores específicos das dietas computados diariamente
Lavanderia	Não se aplica	Não se aplica	Pregões eletrônicos	Valor definito por kit de enxoval. Eram distribuídos seis kits por paciente, com repasse de 80% dos valores dos kits à empresa prestadora do serviço

^a^Sesab: Secretaria de Saúde da Bahia; ^b^SIGTAP: Sistema de Gerenciamento da Tabela de Procedimentos, Medicamentos e OPM do SUS; ^c^AIH: autorização de internamento hospitalar; ^d^UTI: unidade de terapia intensiva.

Para as estimativas de custos assistenciais, utilizou-se uma abordagem *bottom-up* (microcusteio), com a identificação, por participante, de cada recurso utilizado. Os recursos utilizados ambulatorialmente foram obtidos no sistema de informação hospitalar SmartHealth, e os hospitalares, no Sistema Gerador do Movimento das Unidades Hospitalares. Todos os custos foram levantados, conforme taxas atualizadas, de acordo com os valores em reais praticados em julho de 2022. 

### Tamanho do estudo

Do total de 68 pacientes inscritos no centro de referência, 66 (97%) foram incluídos. Dois casos não foram incluídos por terem menos de duas consultas de seguimento, à época do término do estudo. 

### Operacionalização das variáveis e métodos estatísticos

Os dados foram coletados em formulários padrões, elaborados no Google Forms, planilhados no Microsoft Excel, e analisados no pacote estatístico R para Windows 4.2.2 e Stata para Mac 18.0.

Para calcular os custos totais, multiplicou-se o custo unitário de um tratamento pela quantidade utilizada, obtendo-se uma estimativa do custo médio do tratamento. Estimou-se o custo médio direto para os custos com medicamentos utilizados no internamento, rouparia e dieta. Os custos apurados foram expressos em unidade monetária (reais), em valores totais, média e desvio-padrão e por pessoa-ano. 

Os custos foram descritos por tipo (medicamento, consultas, exames, dieta, rouparia), por local (internamento, hospitalar e ambulatorial) e por período (2005-2022 e 2013-2022). Esta última categoria foi utilizada porque os participantes com diagnóstico precoce só iniciaram acompanhamento após a implantação da triagem neonatal na Bahia em 2013 (5,13).

Foram descritas média e desvio-padrão, para as variáveis contínuas com distribuição normal, e mediana e intervalo interquartílico, para as não normais. Para a comparação de proporções e de medianas entre os com diagnóstico tardio e precoce, utilizaram-se o teste do qui-quadrado ou exato de Fisher, quando indicado, e o teste não paramétrico U de Mann-Whitney. Utilizou-se o teste de correlação de Spearman para analisar associação entre as variáveis tempo de seguimento e custo. A força da correlação foi classificada como previamente descrito (16). Dado a diferença de tempo de participação na coorte entre aqueles com diagnóstico precoce e tardio, a densidade de internamentos foi calculada e expressa em 100 pessoas-ano. 

Em todas as análises, os testes bicaudais e os valores de p (p-valor) menores ou iguais a 0,05 foram considerados estatisticamente significantes.

## Resultados 

Foram avaliados 66 participantes, sendo 47 (71,2%) com diagnóstico tardio, obtidos a partir de 2005, e 19 com diagnóstico precoce (28,8%), tendo nascidos entre 2013 e 2021, sendo o sexo masculino predominante (56,1%) (Tabela 2). A média (desvio-padrão) de idade à admissão na coorte foi 52,7 (±44,2) meses. O diagnóstico pela triagem neonatal foi mais precoce em relação àqueles com diagnóstico tardio (3,4±0,3 versus 72,0±10,4 meses; p-valor<0,001).

**Tabela 2:** Número de participantes com fibrose cística, de acordo com as características sociodemográficas e clínicas e com a condição de diagnóstico de hospital universitário. Salvador, 2005-2022 (n=66)

Variável	Total (n=66)	Diagnóstico precoce (n=19)	Diagnóstico tardio (n=47)
	n (%)	n (%)	n (%)
**Faixa etária no final coorte** (anos)			
0-9	37 (56,9)	19 (100,0)	18 (39,1)
10-19	21 (32,3)	-	21 (45,7)
≥20	7 (10,8)	-	7 (15,2)
Sexo			
Feminino	29 (43,9)	9 (47,4)	20 (42,6)
Masculino	37 (56,1)	10 (52,6)	27 (57,4)
**Raça/cor da pele**			
Branca	11 (16,7)	3 (15,8)	8 (17,0)
Parda	48 (72,7)	14 (73,7)	34 (72,3)
Preta	3 (4,5)	1 (5,3)	2 (4,2)
Não declarado	4 (6,1)	1 (5,3)	3 (6,4)
**Necessidade de internamento**			
Não	24	6 (31,6)	18 (38,3)
Sim	42	13 (68,4)	29 (61,7)
Óbito			
Não	62 (94,0)	17 (69,7)	45 (24,2)
Sim	4 (6,0)	2 (3,0)	2 (3,0)

O tempo mediano (p25; p75) de seguimento foi 95,9 (53,8; 173,9) meses, variando de 7,4 a 279,0 meses. Participantes com diagnóstico precoce tiveram um tempo total acumulado de seguimento, em anos, significativamente menor, comparado aos com diagnóstico tardio (69,9 meses vs. 550,8 meses; p-valor<0,001).

Identificaram-se 121 episódios de internamentos: 96 (79,3%) ocorreram entre participantes com diagnóstico tardio; destes, 61,7% (29/47) internaram. Foram internados 68,4% (13/19) daqueles com diagnóstico precoce em algum momento do seguimento. Aqueles com diagnóstico precoce foram hospitalizados, predominantemente, no primeiro ano de vida (88,0%) e com duração mediana similar àqueles com diagnóstico tardio. A densidade de internamento (por 100 pacientes-ano) foi 18,0 para aqueles com diagnóstico tardio e 34,3 para aqueles com diagnóstico precoce (p-valor 0,008). A mediana (p25; p75) do número de internamentos entre aqueles com diagnóstico precoce e tardio não diferiu (1,0 [0,0; 2,0]; amplitude: 0-6 vs. 2,0 [0,0; 4,0]; amplitude: 0-11; p-valor 0,347).

No período 2005-2022, o custo total (diagnóstico e tratamento) para todos os 66 participantes, na perspectiva do SUS, foi R$ 6.702.599,30, perfazendo o gasto médio por paciente-ano de R$ 10.731,82. Observaram-se valores de R$ 7.294,77 por paciente-ano para aqueles com diagnóstico precoce e R$ 11.167,45 por paciente-ano para aqueles com diagnóstico tardio, com razão de custo de 1,5 vez maior para os pacientes com diagnóstico tardio (p-valor<0,001).

Os custos diretos com medicamentos representaram 96,9% dos gastos totais, cujo valor global foi R$ 6.497.624,07 (Tabela 3). A alfadornase foi o medicamento com os maiores dispêndios e com valor mais elevado entre aqueles com diagnóstico tardio (R$ 8.594,85 paciente-ano), comparado àqueles com diagnóstico precoce (R$ 3.253,43; p-valor<0,001) (Tabela 3). Os gastos por pessoa-ano para aqueles com diagnóstico tardio, computados entre 2013 e 2022, não foram diferentes para o período total (2005-2022), exceto pelo maior valor para a tobramicina (R$ 1.195,80 vs. R$ 554,24; p-valor<0,001), consultas ambulatoriais (R$ 82,26 vs. R$ 57,58; p-valor<0,001), exames ambulatoriais (R$ 81,49 vs. R$ 54,83; p-valor<0,001), medicamentos durante o internamento (R$ 159,32 vs. R$ 69,97; p-valor<0,001) e dieta durante o internamento (R$ 431,08 vs. R$ 272,09; p-valor<0,001) (Tabela 3) 

**Tabela 3:** Valores totais (R$), médios e desvio-padrão (R$) e por pessoa-ano (R$) dos custos ambulatoriais e hospitalares, segundo tipo, e razão de densidades de incidências (RDI) dos custos por pessoa-ano, entre os indivíduos de diagnóstico precoce e diagnóstico tardio, estratificado por período de análise (2005-2022 e 2013-2022), sob as perspectivas do Sistema Único de Saúde e da instituição de saúde. Salvador, 2005-2022 (n=66)

Custos em R$ (tipo)	Todos os casos (n=66)	2005-2022				RDI	Todos os casos (n=66)	2013a-2022				RDI
		n	Diagnóstico tardio	n	Diagnóstico precoce			n	Diagnóstico tardio	n	Diagnóstico precoce	
Ambulatorial												
Medicamentos^c^		45		19				17		19		
Valor total	6.497.624,07		6.042.576,53		455.047,54		1.666.038,00		1.210.990,00		455.047,50	
Média±DP^b^	96.976,19 ±108.553,22		128.560,79 ±113.883,41		23.949,87 ±36.149,47		45.028,05 ±60.939,39		71.234,72 ±74.171,83		23.949,87 ±36.149,47	
Pessoa-ano	10.403,28		10.902,01		6.471,91	0,59	9.197,49		10.926,63		6.471,91	0,59
Alfadornase^c^		37		6				9		6		
Valor total	4.992.384,37		4.763.630,92		228.753,45		1.114.468,75		885.715,30		228.753,45	
Média±DP	74.513,20 ±89.986,39		101.353,85 ±93.799,57		12.039,66 ±29.949,90		30.120,78 ±51.375,82		52.100,90 ±63.019,42		12.039,66 ±29.949,90	
Pessoa-ano	7.993,52		8.594,85		3.253,43	0,38	6.152,51		7.991,71		3.253,43	0,41
Tobramicina^c^		34		14				14		14		
Valor total	535.176,20		472.787,70		62.388,50		194.917,60		132.529,10		62.388,50	
Média±DP	7.987,70 ±12.480,27		10.059,31 ±14.207,56		3.283,60 ±4.141,33		5.268,04 ±7.370,45		7.795,83 ±9.472,79		3.283,60 ±4.141,33	
Pessoa-ano	624,55		554,24		887,31	1,04	1.076,05		1.195,80		887,31	0,74
Pancreatina^c^		41		18				17		18		
Valor total	970.063,50		806.157,91		163.905,59		356.651,30		192.745,80		163.905,59	
Média±DP	14.475,29 ±14.876,52		17.147,63 ±15.663,63		86.262,61 ±10.866,23		9.639,23 ±10.217,38		11.337,99 ±9.589,75		8.6262,61 ±10.866,23	
Pessoa-ano	1.552,86		1.454,13		2.331,15	1,60	1.968,93		1.739,13		2.331,15	1,34
Consultas^c^		47		19				17		19		
Valor total	37.876,50		31.913,40		5.963,40		15.080,80		9.117,40		5.963,40	
Média±DP	563,33 ±394,50		679,01 ± 400,96		313,86 ±203,83		443,55 ±235,18		536,32 ±250,63		313,86 ±203,83	
Pessoa-ano	60,64		57,58		84,81	1,47	83,25		82,26		84,81	1,03
Exames^c^		45		19				16		19		
Valor total	40.317,03		30.391,15		9.925,88		18.958,08		9.032,20		9.925,88	
Média±DP	601,75 ±431,77		646,62 ±488,52		522,41 ±204,05		512,38 ±251,30		531,31 ±279,89		522,41 ±204,05	
Pessoa-ano	64,55		54,83		141,17	2,57	104,66		81,49		141,17	1,73
Hospitalar												
Internamentos^c^		27		13				11		13		
Valor total	126.781,40		84.813,64		41.967,77		73.452,58		31.484,81		41.967,77	
Média±DP	1.173,90 ±1.478,33		1.804,55 ±2.670,26		2.208,83 ±4.447,06		1.985,20 ±3.494,46		1.850,05 ±2.214,11		2.208,83 ±4.447,06	
Pessoa-ano	203,00		153,03		596,89	3,91	405,50		284,09		596,89	2,10
Medicamentos^d^		26		12				11		12		
Valor total	58.473,07		38.782,71		19.690,36		37.347,57		17.657,21		19.690,36	
Média±DP	872,73 ±2.215,72		825,16 ±2.113,35		1.036,33 ±2.551,68		1.009,39 ±2.358,46		1.038,66 ±2.263,81		1.036,33 ±2.551,68	
Pessoa-ano	93,62		69,97		280,04	4,00	206,18		159,32		280,04	1,76
Dieta^d^		27		13				11		13		
Valor total	191.621,70		150.804,70		40.816,98		88.592,58		47.775,60		40.816,98	
Média±DP	2.860,03 ±4.138,92		3.208,61 ±4.693,54		2.148,26 ±2.282,31		2.394,39 ±2.694,80		2.810,33 ±3.140,90		2.148,26 ±2.282,31	
Pessoa-ano	306,81		272,09		580,52	2,13	489,08		431,08		580,52	1,35
Lavanderia^d^		27		13				11		13		
Valor total	161.924,60		141.925,70		19.998,91		62.811,08		42.812,17		19.998,91	
Média±DP	2.416,78 ±4.121,07		3.019,69 ±4.728,59		1.052,57 ±1.375,72		1.697,60 ±2.471,87		1.052,57 ±1.375,72		1.052,57 ±1.375,72	
Pessoa-ano	259,27		256,07		284,43	1,11	346,75		386,29		284,43	0,74

^a^Ano da implantação da triagem neonatal para a fibrose cística na Bahia, havendo os custos dos triados levantados a partir desse período; ^b^DP: desvio-padrão; ^c^Componentes de custos que compreendem a perspectiva do Sistema Único de Saúde; ^d^Componentes de custos que compreendem a perspectiva da instituição de saúde.

Os custos ambulatoriais com consultas e exames diagnósticos foram R$ 80.533,10 (1,2% do total) (Tabela 3). Registraram-se 5.029 consultas no centro de referência, custo por paciente entre R$ 22,60 e R$ 1.391,20; média de R$ 565,00±R$ 371,00. Foram realizados 215 exames de imagem em regime ambulatorial e 136 durante os internamentos. Realizaram-se 1.491 exames de bacteriologia respiratória, custo total de R$ 15.282,75 (Tabela 4). 

**Tabela 4:** Quantitativo dos componentes de custos de recursos de saúde, utilizados na assistência aos indivíduos com fibrose cística e seus custos unitários e totais sob a perspectiva do Sistema Único de Saúde no hospital universitário. Salvador, 2005-2022 (n=66)

Componentes dos custos	Custo unitário (R$)	n	Custos totais (R$)
Ambulatoriais			
Consultas médicas	10,00	1.674	16.740,00
Consultas profissionais não médicos	6,30	3.355	21.136,50
**Exames de apoio diagnóstico** (ambulatoriais)			
Avaliação da função pulmonar	9,14	25	228,50
Dosagem do cloreto do suor	150,00	61	9.150,00
Ecocardiograma	67,86	1	67,86
Endoscopia digestiva	63,31	6	379,86
Exame de bacteriologia	10,25	1.491	15.282,75
Outras tomografias	86,75	12	1041,00
Outras ultrassonografias	24,20	11	266,20
Radiografia de abdome	10,73	1	10,73
Radiografia de tórax	9,50	91	864,50
Ultrassonografia de abdome	37,95	61	2.314,95
Ultrassonografias com Doppler	39,60	4	158,40
Tomografia de tórax	136,41	25	3.410,25
Tomografia de abdome	277,26	3	831,78
Hospitalares^a^			
Internamentos		121	126.781,41

^a^Nos custos dos internamentos, estão incluídos os exames que têm valores em reais repassados pelo Sistema Único de Saúde.

O valor total reembolsado pelo SUS com a realização da dosagem de cloreto do suor foi R$ 9.150,00 (Tabela 4). Entre a primeira e a segunda dosagem de cloreto do suor, houve 112 exames realizados na instituição. O SUS repassou para a instituição os valores monetários de até 2 dosagem de cloreto do suor partir de 2013, o que representou 54,5% do total de exames realizados. Oito indivíduos realizaram a primeira dosagem de cloreto do suor em outra instituição, não sendo contabilizados. 

Na perspectiva do estabelecimento de saúde, os custos totais durante os internamentos com medicamentos, dieta e lavanderia foram R$ 412.019,37. Na perspectiva do SUS, os repasses para os internamentos foram R$ 126.781,40, perfazendo 30,8% do total (Tabela 4). Não houve diferença estatisticamente significante nesses custos, segundo o diagnóstico tardio ou precoce (p-valor 0,416). Quanto aos medicamentos prescritos durante os internamentos, não houve diferença dos gastos médios (±desvio-padrão) dos internamentos para aqueles com diagnóstico precoce e tardio por indivíduo (R$ 825,16±R$ 2.113,35 vs. R$ 1.036,33± R$ 2.551,68; p-valor 0,681).

Houve correlação fraca entre os custos totais e o maior tempo de seguimento tanto para o grupo com diagnóstico tardio (rhô=0,324) quanto para aqueles com diagnóstico precoce (rhô=0,313) (Figura 1).

**Figura 1:** Correlação entre os custos totais e o tempo de seguimento para os grupos de participantes de diagnóstico tardio (n=47) e triados (n=18) no hospital universitário. Salvador, 2005-2022

Houve a perda de 10 participantes, sendo 1 por transferência para outro centro de referência, 4 por óbito e 5 por perda de seguimento. A comparação desses 10 participantes com os demais não revelou diferença significante nos tempos de seguimento (10,8±2,7 vs. 9,2±0,8; p-valor 0,654) e de internamento (18,1±9,4 vs. 29,4±5,5; p-valor 0,125). Os custos totais (R$ 29.550,30±R$ 46.748,88 vs. R$ 113.741,10±R$ 114.722,50; p-valor 0,003) e por pessoa-ano (R$ 3.143,96±R$ 2.602,54 vs. R$ 11.884,47±R$ 10.227,07; p-valor 0,005) foram significativamente menores entre as perdas de seguimento.

Não se mensuraram os custos de 14 internações de 7 participantes devido à ausência de informações detalhadas nos registros do SUS. Não foi possível quantificar os medicamentos utilizados por 6 participantes com diagnóstico tardio entre 2005 e 2011, por não serem distribuídos pela instituição à época. Os demais custos relacionados a esses indivíduos foram incluídos.

## Discussão 

Por meio dos dados, notou-se que expressiva quantia foi investida pelo SUS no cuidado ao indivíduo com fibrose cística no período 2005-2022. Observou-se que os medicamentos alcançaram 97% do total de gastos, havendo menor gasto por pessoa-ano entre aqueles com diagnóstico precoce, na razão de 0,59 (medicamentos), basicamente pelo menor uso de alfadornase, porém maior razão para gastos com exames (1,73) e internamentos (2,10). 

Os dados evidenciaram o compromisso do SUS com o cuidado integral da fibrose cística, mas indicaram a necessidade de revisar a alocação de recursos, especialmente diante do alto custo dos medicamentos. O maior uso de exames e internações em casos diagnosticados precocemente sugeriu acompanhamento inicial mais intensivo, que pode evitar complicações futuras. Isso reforça a importância de políticas de detecção precoce e cuidado integrado, visando à sustentabilidade do sistema e à melhor qualidade de vida dos sujeitos.

Este estudo teve algumas limitações. O caráter unicêntrico e a predominância de participantes menores de 18 anos limitaram a validação externa dos resultados. Entretanto, em 2021, 75% das pessoas com fibrose cística no Brasil estavam nessa faixa etária (4). Por se tratar de uma doença rara no país, estudos de análise de custos são escassos, pois as instituições de saúde, principalmente as públicas, não acompanham os seus custos e não possuem sistemas informatizados para registros dos gastos na assistência à saúde prestada (17). A instituição não possuía centro de custo para materiais descartáveis por indivíduo, o que impossibilitou o levantamento completo dos insumos utilizados e dos custos fixos. 

Custos indiretos e de outras instituições também não foram incluídos, o que limitou a generalização dos achados, podendo levar à subestimação do custo médio anual por paciente. Os valores obtidos não foram validados com outros hospitais ou fontes externas. De fato, medicamentos adquiridos em pregões podem apresentar grandes diferenças de valores na dependência da quantidade adquirida, do escopo do comprador (secretaria de saúde, unidade de saúde, governo federal) (18). 

Outra limitação correspondeu ao fato de os custos por pessoa-ano não terem sido ajustados pela gravidade da doença e idade do diagnóstico, potenciais confundidores (19). Os dados de gravidade da doença não foram aferidos neste estudo, sendo essa uma recomendação para futuros estudos nessa temática.

Estudos anteriores evidenciaram que os medicamentos correspondiam à maior parcela dos custos associados ao tratamento das manifestações clínicas da doença, reflexo da dependência de terapias contínuas e de alto valor (20-23). Independentemente das diferenças metodológicas entre os estudos, há consistência quanto ao padrão de distribuição dos gastos, sendo os medicamentos e as internações os principais componentes do custo total (6,23,24). Esse perfil de custo tende a se intensificar com a progressão da doença, o que é esperado em condições crônicas de alta complexidade terapêutica (22). No entanto, a comparabilidade entre os achados é limitada pelas diferenças nos protocolos clínicos adotados, no acesso às tecnologias de saúde e nas metodologias empregadas para estimativa de custos em diferentes contextos nacionais (7,19,24).

Neste estudo, a alfadornase foi o medicamento de maior impacto no custo de paciente-ano, embora significativamente menor entre os triados, uma vez que nesses casos é utilizado apenas quando há potencial de benefício para a função pulmonar ou risco de ocorrência de infecção das vias respiratórias inferiores (14,26). Esse achado reforça o já descrito previamente, de que pior função pulmonar está associada ao maior custo (19). Considerando apenas o período 2013-2022, o custo com o uso de tobramicina foi 35% maior para aqueles com diagnóstico tardio neste estudo.

Espera-se que o tratamento precoce e otimizado possa reduzir custos, ao minimizar a progressão da doença. Menor demanda para uso de medicamentos de alto custo, evitar internamentos prolongados, com uso de antimicrobianos e oxigenoterapia, corroboram essa hipótese (27). Isso reforça o papel da triagem neonatal na redução de custos e maximização de resultados. 

Estudos prévios podem explicar o menor custo de paciente-ano observado entre aqueles com diagnóstico precoce. Na França, foram acompanhadas, por 10 anos, 779 crianças com fibrose cística nascidas entre 2006 e 2011. Observou-se que, no primeiro ano de vida, ocorreu menor custo médio por paciente entre os precoces, comparados ao com diagnóstico tardio, baseado em sintomas (€ 12.056,00±€ 10.073,00 vs. € 3.861,00±€ 17.493,00) (19). 

Identificaram-se três perfis de custo, sendo a maioria daqueles com diagnóstico precoce classificados no perfil “baixo e estável” (19). Isso reforça que fatores confundidores, não avaliados neste estudo, podem tornar essa equação ainda mais vantajosa. Possivelmente, se a confirmação diagnóstica e o início do tratamento para os casos com diagnóstico precoce fossem ainda mais cedo do que a mediana aqui descrita, grande parte dos internamentos dessas crianças – que neste estudo foi responsável por quase nove entre dez internamentos no primeiro ano de vida – poderia ser evitada, reduzindo o custo e, provavelmente, a morbidade futura dos casos. Com relação ao observado na França, o perfil de custos “altíssimo e crescente” se associou com pior função pulmonar, desnutrição e mais infecções por *Pseudomonas aeruginosa* (19).

Os internamentos por fibrose cística ocorrem, principalmente, por infecções respiratórias, tratadas de modo semelhante entre os grupos precoce e tardio, o que contribui para custos comparáveis. Entretanto, a menor duração do seguimento nos pacientes diagnosticados precocemente e a concentração de hospitalizações no primeiro ano sugerem um possível efeito coorte, o qual pode mascarar benefícios econômicos da triagem neonatal, que tenderiam a se evidenciar em seguimentos mais longos, com menor frequência de internações decorrente da menor gravidade clínica. Estudos confirmaram que os custos hospitalares se associaram à gravidade da doença. No Canadá, em 2021, indivíduos com diagnóstico precoce apresentaram melhores desfechos clínicos e menor necessidade de hospitalização (28), achado não reproduzido nesta análise.

Embora o diagnóstico precoce seja essencial para reduzir morbimortalidade, ele não garante menor custo assistencial, especialmente nas formas graves. É importante fortalecer não apenas a triagem neonatal, mas também o fluxo de encaminhamento e o início mais precoce do tratamento especializado, a fim de ampliar os benefícios clínicos e econômicos (13,27). Esse aprimoramento independe da incorporação dos novos moduladores da proteína, que foram aprovadas para o uso acima dos 6 anos de idade (10). A eficiência dos gastos no diagnóstico e tratamento da doença depende da incorporação de novos medicamentos e da melhora da performance da triagem neonatal (13).

Não se observaram diferenças estatísticas entre as médias de custos de internamentos comparando indivíduos com diagnóstico precoce e tardio. A densidade de internamentos foi maior entre os casos com diagnóstico precoce possivelmente devido ao menor tempo de acompanhamento dessas crianças na coorte e à identificação precoce de casos mais graves, que poderiam não sobreviver sem a triagem neonatal. 

Levantou-se a hipótese de que o diagnóstico precoce da doença pode ter identificado casos de maior gravidade que poderiam falecer antes do diagnóstico clínico, na ausência da triagem neonatal. Neste estudo, 38,9% das crianças diagnosticadas precocemente tiveram internações muito prolongadas no momento do diagnóstico, o que corroborou a gravidade clínica e um potencial atraso no diagnóstico, mesmo em crianças triadas. Na Bahia, contatou-se atraso no diagnóstico da fibrose cística pela triagem neonatal, com mediana de idade ao diagnóstico de 3,5 meses (13). Essa combinação de fatores deve ter maximizado a densidade de internamentos e contribuiu para as internações prolongadas entre aqueles com diagnóstico precoce, um viés de coorte. Análises futuras dessa mesma coorte, considerando ocorrência de estabilidade clínica, obtida com o seguimento adequado e refletida em ausência de internamentos, deverão resultar em inversão destes achados.

A análise, sob a perspectiva da instituição de saúde, demonstrou percentual de repasse insuficiente pelo SUS para a cobertura dos gastos com as hospitalizações, contemplando-se os exames de análises clínicas, medicamentos, dietas e rouparia, para os quais não há repasse direto (29), independentemente do tipo e da quantidade de exames executados, não refletindo, portanto, os custos reais dos internamentos. Isso revela que o uso racional dos recursos pelas equipes assistenciais e a maior eficiência na gestão assistencial nos hospitais que atendem às demandas desses indivíduos devem ser maximizados (24,30). Também explicita que análises de custos utilizadas em modelos econômicos, sob a perspectiva do SUS, minimizam os custos, pois todo o montante financeiro que não foi repassado para ao hospital é financiado com recursos públicos. 

O estudo demostrou que o uso do microcusteio permitiu identificar com precisão os principais componentes do custo da fibrose cística, especialmente o peso dos medicamentos. O levantamento detalhado se aproximou do custo real para a instituição, o que evidenciou a insuficiência dos repasses públicos e a necessidade de maior eficiência na alocação de recursos (23;28-30). A triagem neonatal, quando bem executada, mostrou-se economicamente vantajosa ao reduzir complicações e custos associados. 

Os dados analisados indicaram benefícios econômicos e sociais da triagem, como a redução do sofrimento familiar e da jornada diagnóstica. Pacientes com diagnóstico tardio apresentaram maiores custos diretos por pessoa-ano, especialmente com medicamentos para quadros mais graves. Os achados deste estudo reforçaram a necessidade de revisão dos repasses do SUS e da eficiência na gestão dos programas públicos.

## Data Availability

Dados disponíveis mediante solicitação ao autor correspondente.
